# Structural metamorphosis and photophysical properties of thermostable nano- and microcrystalline lanthanide polymer with flexible coordination chains

**DOI:** 10.1080/14686996.2023.2183711

**Published:** 2023-03-03

**Authors:** Takayuki Nakanishi, Yuichi Hirai, Jian Xu, Takashi Takeda, Shunsuke Watanabe, Atsuo Yasumori, Shou Hakamada, Yuichi Kitagawa, Yasuchika Hasegawa

**Affiliations:** a Research Center for Functional Materials, National Institute for Materials Science (NIMS), Tsukuba, Ibaraki, Japan; b International Center for Young Scientists (ICYS), National Institute for Materials Science (NIMS), Tsukuba, Ibaraki, Japan; c Department of Materials Science and Technology, Tokyo University of Science, Tokyo, Japan; d Faculty of Engineering, Hokkaido University, Sapporo, Japan; e Institute for Chemical Reaction Design and Discovery (WPI-ICReDD), Hokkaido University, Sapporo, Japan

**Keywords:** Coordination polymers, luminescence property, europium, phosphor

## Abstract

Luminescent lanthanide coordination polymer crystals (LCPCs) represent an area of growing interest in materials chemistry owing to their unique and tailorable functional properties. The LCPCs provide a high level of structural tunability, including size- and morphology-dependent properties; therefore, they are promising materials for next-generation phosphors in a wide range of applications such as light emitting diodes. Here, by controlling the morphology of thermostable europium coordination polymer crystals, [Eu(hfa)_3_(dpbp)]_n_, hfa: hexafluoroacetylacetonate and dpbp:4,4’-bis(diphenyl　phosphoryl) biphenyl), we realized a novel red phosphor with narrow linewidth emission (FWHM = 7.8 nm). The obtained luminescent LCPCs with unique structures were characterized by X-ray diffraction (XRD), scanning transmission electron microscopy (STEM), dynamic light scattering (DLS) and thermogravimetric analysis. Among, them, size tunable crystalline polymer spheres were found to have high internal quantum efficiency (*ex*., IQE = 79%) and highly thermostability (>300°C), and to exhibit dispersibility in PMMA media. The obtained results on the structural tunability of these materials can be used for the development of synthesis techniques for nanoscale materials based on crystalline lanthanide-based coordination phosphors.

## GRAPHICAL ABSTRACT

**Figure:**
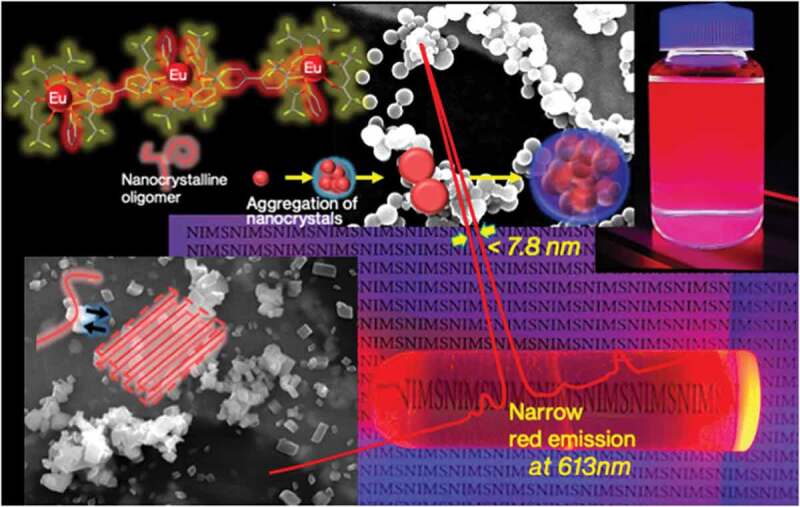

## 1. Introduction

Thermally and chemically stable luminescent lanthanide coordination polymer crystals (LCPCs) are novel candidate phosphor materials for various luminescence applications, such as light emitting diode (LED) displays [1,2], security inks [3], and wavelength converters for solar cells [4–6]. In particular, they are expected to play an active role as advanced nanoscale high-brightness phosphors that can replace quantum dots containing harmful elements in micro-LED phosphor applications for next-generation displays [7]. Previously, we proposed thermostable europium coordination polymer crystals composed of europium ions and organophosphine oxides (decomposition temperature>280°C) as a new series of red or green phosphor materials with narrow line width emission [8–10]. The crystalline, ceramic-like close-packed structure of LCPCs with CH-π, CH-F, and π-π intra-/intermolecular interactions endows these materials with high thermal stability and a small non-radiative rate constant, *k*_nr_, owing to their low vibrational structure to enable the realization of strong luminescence. Among the various LCPCs, crystalline [Eu(hfa)_3_(dpbp)]_n_ polymers composed of π-conjugated planar bridging ligands are particularly promising LED phosphors that exhibit excellent thermal stability (>300°C) and internal quantum efficiency (>80%) [11,12]. However, in conventional stoichiometric synthesis, LCPCs are obtained as micro-sized crystalline powder compounds that are insoluble in organic solvents and cannot be used as the desired nanosized phosphors for next-generation displays. We previously reported the use of nanosized [Eu(hfa)_3_(dpbp)]_n_ using a polymerization terminator ligand; unfortunately, the physical strength and luminous properties of these materials could not be preserved [13,14]. If the synthesis of morphology controllable and durable LCPC phosphor is established, it could be a promising nanophosphor as well as the quantum dot series [15].

**Figure 1.**
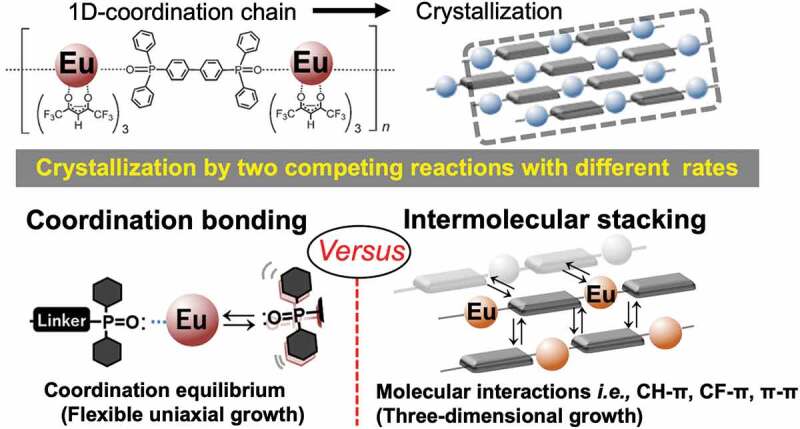
Crystallization process of thermostable [Eu(hfa)_3_(dpbp)]_n_ coordination polymer. The polymer crystallization is based on several equilibrium reactions, e.g. coordination- and stacking equilibrium due to molecular interactions such as the CH-π, CF-π, and π-π interactions.

To realize next-generation crystalline coordination phosphors, it is important to understand the fundamental morphological properties of LCPCs and elucidate their optical properties. Figure 1 illustrates the crystallization process of a specific [Eu(hfa)_3_(dpbp)]_n_ polymer using two or more equilibrium reactions, that is, the coordination- and stacking-equilibrium due to molecular and CH–π and CF–π, interactions. These intricate crystal growth mechanisms in organic/inorganic coordination polymers offer new possibilities in materials chemistry. Mirkin et al. reviewed the structural tailorabilities of transition metal coordination polymers and the functionality of different synthesis solvents or metal species, revealing the degrees of freedom in material design [16,17]. Here, as a simple and effective method, that is, the controllability of the LCPC morphology by changing the amounts of chemicals and organic solvents used during lanthanide coordination formation, is demonstrated for the first time. The obtained coordination compounds with unique structures are characterized via X-ray diffraction (XRD), scanning transmission electron microscopy (STEM), dynamic light scattering (DLS), and differential scanning calorimetry (DSC) thermogravimetric (DSC-TG) analysis. Their photophysical properties are discussed using emission spectra, emission lifetimes, and quantum efficiency to estimate the radiative/non-radiative rate constants. In conclusion, we successfully demonstrate morphology-controllable crystalline [Eu(hfa)_3_(dpbp)]_n_ polymers and discuss their formation mechanism. This study provides important information on the detailed reaction processes and functionalization of lanthanide coordination polymers. Progress in the field of the design of new materials using coordination polymers that will play an important role in next-generation phosphor materials is expected to contribute to the improvement in structural selectivity and optical functionality.

## 2. Experimental section

### 2.1. Preparations of [Eu(hfa)_3_dpbp]_n_ with various morphological changes

**Figure 2.**
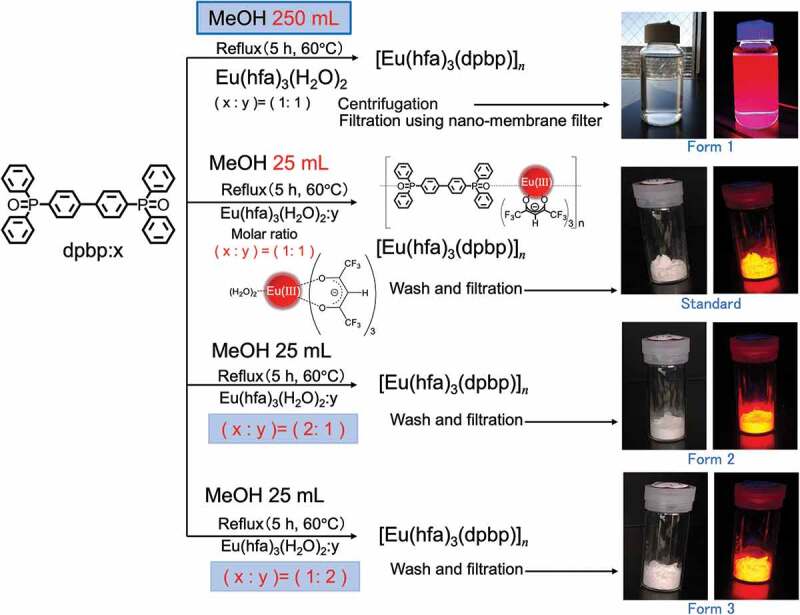
Four synthesis schemes of europium coordination polymers, [Eu(hfa)_3_(dpbp)]_*n*_; Standard (stoichiometric synthesis), Form 1 (dilute solvent, 250 mL MeOH), Form 2 (linker amount twice larger than the stoichiometric amount), and Form 3 (europium amount twice larger than the stoichiometric amount).

The samples were prepared under four different conditions using the method described below: Table 1 lists the synthesis conditions for the [Eu(hfa)_3_(dpdp)]_n_ coordination compounds. Figure 2 also illustrates each preparation scheme for obtaining europium coordination compounds, [Eu(hfa)_3_(dpbp)]_n_. The synthesis details of the main components, namely the 4,4’-bis(diphenyl phosphoryl) biphenyl (dpbp) linker ligand and Eu(hfa)_3_(H_2_O)_2_, are described in the Supporting Information (SI) and in our previous study [11]. The dpbp (0.40 g, 0.72 mmol) in 15 mL MeOH was added into the solution of Eu(hfa)_3_(H_2_O)_2_ (0.58 g, 0.72 mmol) in 10 mL MeOH. The solution was stirred in a closed system at 60°C for 5 h, and then the obtained white precipitate was filtered and washed with cooled MeOH (~0°C). The obtained white powder sample is named as ‘Standard’. The other samples, named ‘Form 1, 2, and 3’ were obtained by the same method, but using the solvent MeOH amounts, and the mixing ratios of the chemicals presented in Table 1. Form 1 was obtained as a transparent liquid; therefore, the sample was collected by centrifugation and by a rapid filtration technique using a nano-membrane filter.

**Table 1.** Synthesis conditions of the [Eu(hfa)_3_(dpdp)]_n_ coordination compounds.

	X/mmol (Eu(hfa)_3_(H_2_O)_2_)	Y/mmol (dpdp)	Synthesis conditions
(a) Standard	0.72	0.72	Stoichiometric ratio in 25 mL MeOH (Standard)
(b) Form 1	0.72	0.72	Dilute solution in 250 mL MeOH (10 times greater than Standard 25 mL)
(c) Form 2	0.72	1.44	Eu concentration (twice the stoichiometric ratio)
(d) Form 3	1.44	0.72	Linker ligand concentration (twice the stoichiometric ratio)

### 2.2. Characterization methods

The structures of the four obtained types of [Eu(hfa)_3_(dpdp)]_n_ LCPCs were characterized by powder XRD (Smart Lab, Rigaku, Japan) with nickel-filtered Cu Kα1 radiation (1.5406 Å). Simultaneous thermogravimetric and calorific value measurements were performed using an STA 449 F5 Jupiter instrument (TG-DSC, NETZSCH, Germany). The powder morphologies were observed by STEM (HD-2300c, Hitachi, Japan) with energy dispersion X-ray spectroscopy mapping and high-angle annular dark field (HAADF) measurements. The photoluminescence (PL) spectra were measured using a spectrofluorometer (FP-8600, JASCO, Japan) and were calibrated using a standard halogen lamp. PL decay was measured with a HORIBA Jobin Yvon Delta Flex spectrometer and a 375 nm pulsed LED. The 2D/3D confocal fluorescence images (1024 × 512 pixels) of LCPCs are recorded by an inverted microscope (Eclipse Ti2, Nikon, Japan) under 405 nm laser excitation. The monitoring wavelength is set to be 570–620 nm, and the *Z*-step for 3D imaging is 0.1 μm for each confocal panel.

## 3. Results and discussion

### 3.1. Structural variations and thermal stabilities

**Figure 3.**
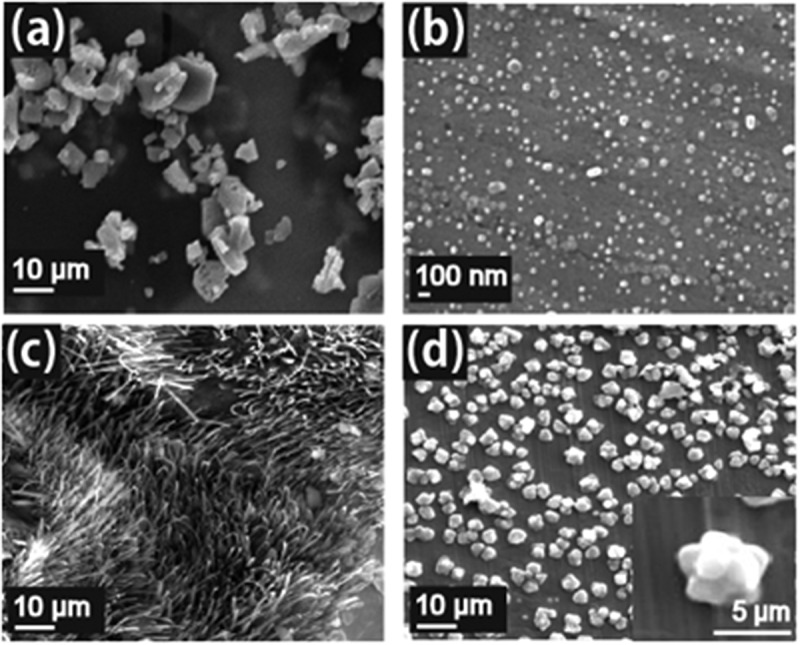
SEM images of LCPCs (a) Standard, (b) Form 1, (c) Form 2, and (d) Form 3.

**Figure 4.**
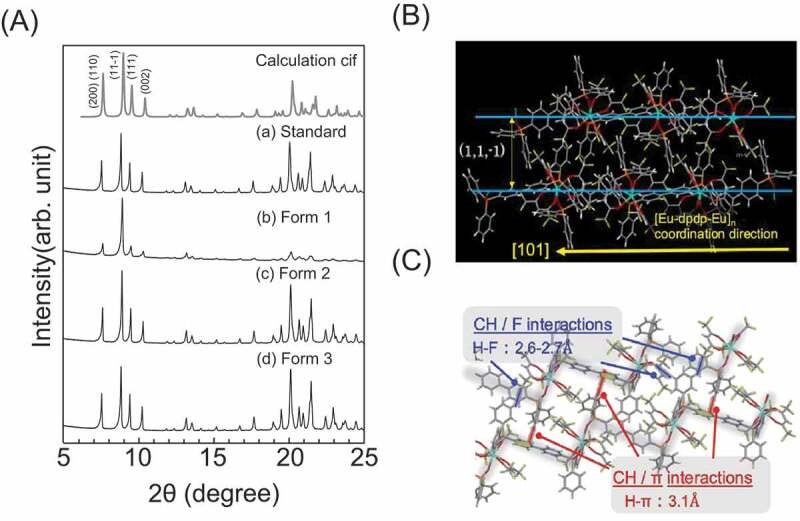
(A) XRD patterns of LCPCs, [Eu(hfa)_3_(dpbp)]_n_; (a) Standard, (b) Sample 1, (c) Sample 2, and d) Sample 3. (B) molecular structure of [Eu(hfa)_3_(dpbp)]_n_ is illustrated using the crystal information file (CIF) [11]. (C) Positions of representative intermolecular interactions (partial, CH/F and CH/π).

As illustrated in Figure 1, the crystallization of crystalline coordination polymers proceeds through the simultaneous occurrence of two or more bonding reactions. The formation of the material with different equilibrium reactions also proceeds at different reaction rates; therefore, it is possible to change the morphological features by varying the conditions such as the amount of the solvent [16]. The shapes of the obtained samples under four different conditions (see Figures 3(a–d)) were observed via field emission scanning electron microscopy (FE-SEM). The SEM image of Standard (Figure 3(a)) shows a thermally stable crystalline coordination polymer with a stoichiometric composition, as reported in our previous study [11,12]. Homogeneous block-shaped particles with the size of several tens of micrometers were dispersed, and their aggregates were observed. On the other hand, well-dispersed spherical particles with sizes of <100 nm were observed in Form 1 (Figure 3(b)). To determine their phase and crystallinity, XRD measurements were performed with the XRD patterns of the LCPCs, [Eu(hfa)_3_(dpbp)]_n_ Standard, Form 1, Form 2, and Form 3 shown in Figures 4(A(a-d)), respectively. The molecular structure from our previous study and positions of representative intermolecular interactions (*ex*., CH/F and CH/π) are illustrated in Figure 4(B,C) using the crystal information file (CIF) of [Eu(hfa)_3_(dpbp)]_n_[See in Ref.11 SI]. All of the obtained samples had the same crystal structure [Eu(hfa)_3_(dpbp)]_n_, and differed only in their orientation and crystallinity. Specifically, the obtained spherical Form 1 particles were oriented in the [11–1] direction. In addition, the high-angle diffraction peaks at approximately 20°, corresponding to the coordination structure around Eu, became weak in the Form 1 sample but were clearly and strongly observed in the Standard. Figure 4(B) and Figure S1 show the relationship between the polymer elongation direction, (Eu-dpdp-Eu)n, [101] and the crystal orientation direction [11–1] of the [Eu(hfa)_3_dpbp]_n_ polymer that was elucidated using crystal information files. In fact, the crystalline spherical particles preferentially grew in the intermolecular stacking direction [11–1], rather than in the Eu polymer elongation direction [11–1]. This means that the process based on the intermolecular interactions proceeds faster than the coordination between Eu and the bridging ligand dpbp under dilute solute synthesis conditions, such as the conditions for Form 1. Moreover, as shown in Figure S2, the average sphere diameter increased with the reaction time (up to 10 h), producing spheres with an average size of around 1 µm. Mirkin et al. [18] described amorphous spheres using Zn-coordination polymers with flexible linear chains that grew to large sizes with increasing reaction time. In our case of highly crystalline lanthanide coordination polymers, owing to strong intermolecular stacking reactions, the nanocrystalline spheres aggregated and grow into large microspheres over time. When the amount of the linker ligand was doubled relative to the stoichiometry of [Eu(hfa)_3_(H_2_O)_2_:dpbp = 1:1], as in Form 2, the morphology was flat and grass-like. A slight orientation in the [11–1] direction is also confirmed by the XRD patterns shown in Figure 4(A(c)). By contrast, when the amount of Eu was doubled relative to the stoichiometry, as in Form 3, the morphology became rock-like, as shown in Figure 3(d).

**Figure 5.**
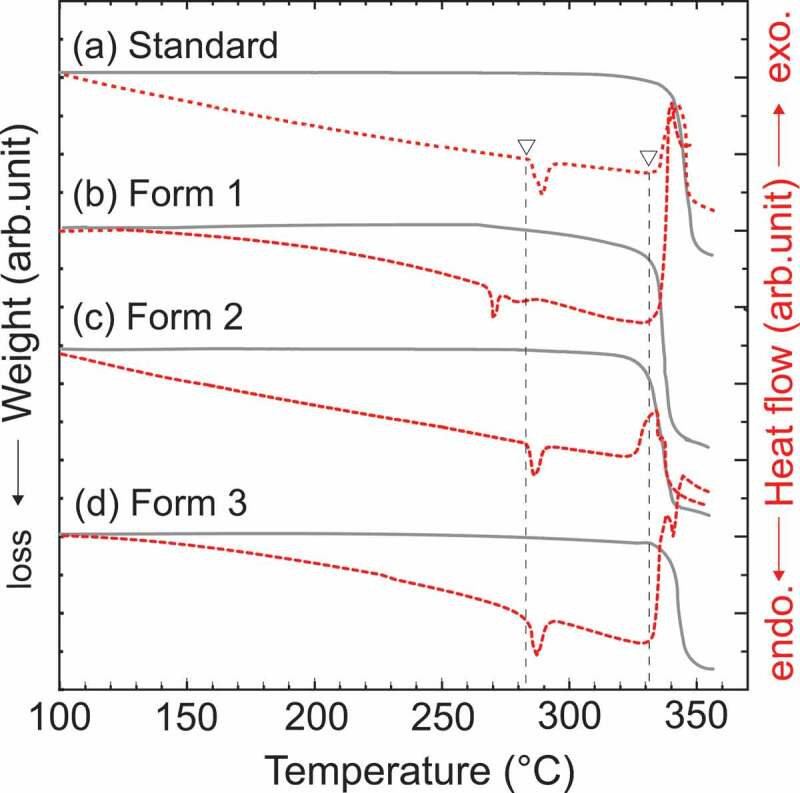
Differential scanning calorimetry (DSC) and thermogravimetry (TG) curves of LCPCs, [Eu(hfa)_3_(dpbp)]_n_; (a) Standard, (b) Form 1, (c) Form 2, and d) Form 3 in nitrogen atmosphere at a heating-rate of 10.0°C/min. Gray solid lines are TG curves, and red dashed lines are DSC curves.

All of the compounds presented here were assigned to the same crystal phase. To evaluate the thermal durability of each sample, DSC and TG curves of the Standard, Form 1, Form 2, and Form 3 Eu coordination polymers are shown in Figures 5(a-d), respectively. The gray solid lines are the TG curves, and the red dashed lines are the DSC curves. The DSC curve of the Standard shows an exothermic peak at approximately 330°C and an endothermic peak at approximately 290°C. Thus, thermogravimetry indicated a rapid decrease in the weight of the sample at 330°C, indicating the combustion of the sample. The endothermic peak observed at approximately 290°C corresponds to the temperature at which the phase transition (See in SI Figure S3, and S4). For the spherical particles in Form 1, an endothermic peak was observed at 270°C, and a rapid mass loss due to combustion was observed at 330°C, similar to the Standard. In conclusion, highly crystalline lanthanide coordination polymers can be structurally tunable leading to the modifications of their size and morphology, while preserving their thermal durability.

### 3.2. Photophysical properties of different formations in [Eu(hfa)_3_dpdp]_n_ polymers

**Figure 6.**
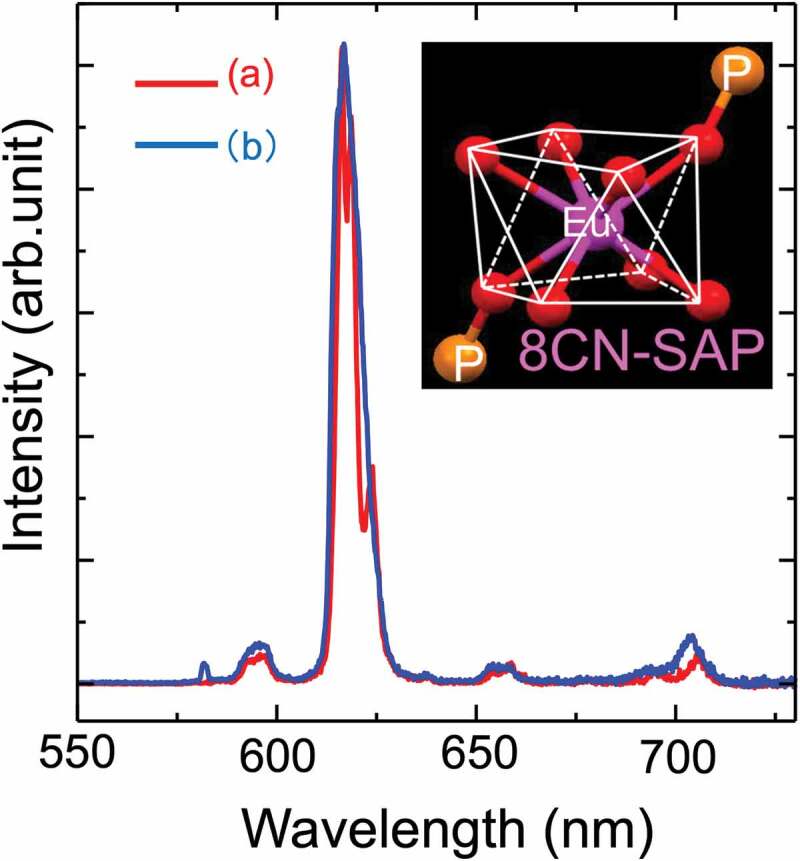
Representative PL spectra of [Eu(hfa)_3_(dpbp)]_n_ coordination polymers: (a) Standard and (b) Form 1 in the solid state. Inset figure shows the coordination geometry around Eu which is categorized as a square antiprism (SAP) structure with the Eu coordination number equal to 8 [11] .

Figure 6 shows representative PL spectra of the Standard and Form 1 Eu(hfa)_3_(dpbp)]_n_ coordination polymers in the solid state. The five characteristic sharp peaks attributed to the ^5^D_0_→^7^F_J_ (J = 0, 1,2, 3, 4) transitions due to the spin-orbit coupling of Eu^3+^ are observed in the range of 550–725 nm. In particular, a main emission band at approximately 613 nm (Full Width Half Maximum<10 nm) is attributed to the ^5^D_0_→^7^F_2_ hypersensitive electronic transitions that are strongly influenced by the coordination structure and split into five energy levels due to crystal fields and Stark splitting [19]. To discuss the difference in their optical properties and local Eu coordination environments, two representative PL spectra of the Standard ‘block-shaped micro-sized crystals’ and the Form 1 ‘spherical nanosized powders’ are shown in Figure 6. The other PL spectra (Form 2 and Form 3) were almost identical to the Standard PL spectrum under excitation at 365 nm (Figure S5). Comparison of the emission spectra reveals a clear difference in the spectral shapes of the hypersensitive J = 2 transition, suggesting that the coordination structure around Eu^3+^ is clearly different despite the similar crystal space group for both Standard and Form 1 samples. This difference may be related to the disorder of the Eu-coordination geometry. J = 0–0 emission at 578 nm, which is strictly forbidden according to the Judd-Ofelt theory [20], was detected only for Form 1, indicating the change of at least the coordination structure to the C_nv_, C_n_, or C_s_ symmetries [19]. The intensity of the J = 0–4 emission band at 690–700 nm reflects that the average covalent intensity in the Judd-Ofelt theory was also increased.

The fundamental photophysical properties of the Standard, Form 1, Form 2, and Form 3 samples are listed in Table 2.

**Table 2.** Photophysical properties of LCPCs, [Eu(hfa)_3_(dpbp)]_*n*_. (a) Standard, (b) Form 1, (c) Form 2, and (d) Form 3.

Sample	*τ*_*obs*_ (ms)	*τ*_*rad*_ (ms)	*Φ*_Ln_ (%)	*k*_*r*_ (s^−1^)	*k*_*nr*_ (s^−1^)
Standard	0.61	0.89	69	1,100	500
Form 1	0.78	0.99	79	1,000	270
Form 2	0.62	0.90	71	1,110	510
Form 3	0.61	0.89	69	1,100	500

These photophysical properties were investigated by estimating the 4f–4f emission quantum yields (Φ_Ln_) and the radiative (*k*_r_) and non-radiative (*k*_nr_) rate constants from the radiative (τ_rad_) and observed lifetimes (τ_obs_). The values were calculated using Werts’ equation [21]. The radiative and observed lifetimes are expressed as

(1) $$\tau_{rad}=\frac{1}{k_{r}}$$

(2) $$\tau_{obs}=\frac{1}{k_{r}+k_{nr}}$$

Φ_Ln_, *k*_r_, and *k*_nr_ are given by

(3) $$\Phi_{Ln}=\frac{k_{r}}{k_{r}+k_{nr}}=\frac{\tau_{obs}}{\tau_{rad}}$$

(4) $$\frac{1}{\tau_{rad}}=A_{MD,0}n^{3}\left(\frac{I_{tot}}{I_{MD}}\right)$$

(5) $$k_{r}=\frac{1}{\tau_{rad}}$$

(6) $$k_{nr}=\frac{1}{\tau_{obs}}-\frac{1}{\tau_{rad}}$$

where A_MD,0_ is the spontaneous emission probability for the ^5^D_0_–^7^F_1_ transition in vacuo (14.65 s^−1^), n is the refractive index [21] (average index of refraction of 1.5 is customarily employed for solid-state materials), and (I_tot_/I_MD_) is the ratio of the total area of the corrected trivalent Eu emission spectrum to the area of the ^5^D_0_–^7^F_1_ band. The lifetimes (τ_obs_) of the Standard and spherical Form 1 samples were estimated as 0.61 ms and 0.78 ms, respectively. The corresponding 4f-4f emission quantum yields (Φ_Ln_) were calculated to be 69% and 79%, respectively. We found that the internal quantum efficiency Φ_Ln_ of crystalline spherical particles increased by approximately 10% as solid-state. This was due to the difference in the coordination geometry of the eight oxygen atoms around Eu (i.e. coordination number (CN) = 8). Thus, nanosized LCPC (Form 1) has a long-period structure (crystallinity) similar to that of [Eu(hfa)_3_dpdp]_n_ (Standard) crystals, but the Eu local environment is different. In addition, a large change point in the photophysical value is a decrease in the non-radiative rate constant: k_nr_ from 510 s^−1^ to 270 s^−1^. Although it cannot be determined because the Eu coordination geometry also changed, we consider that the ^5^D_0_-^7^F_4_ emission, which is proportional to the strength of electronegativity [19], has become strong, resulting in a lower vibrational structure. It was concluded that nanosized spheres with excellent optical properties were obtained while maintaining high thermal stability. By contrast, LCPCs with the same structure and spectral shape as found in the Standard, Form 2, and Form 3 samples do not show large differences in their Φ_Ln_ and lifetimes. This result shows that the difference in stoichiometry during the synthesis affects the shape of the compound, but not its photophysical properties.

### 3.3. Formation mechanism of different forms in crystalline [Eu(hfa)_3_dpdp]_n_ polymers

**Figure 7.**
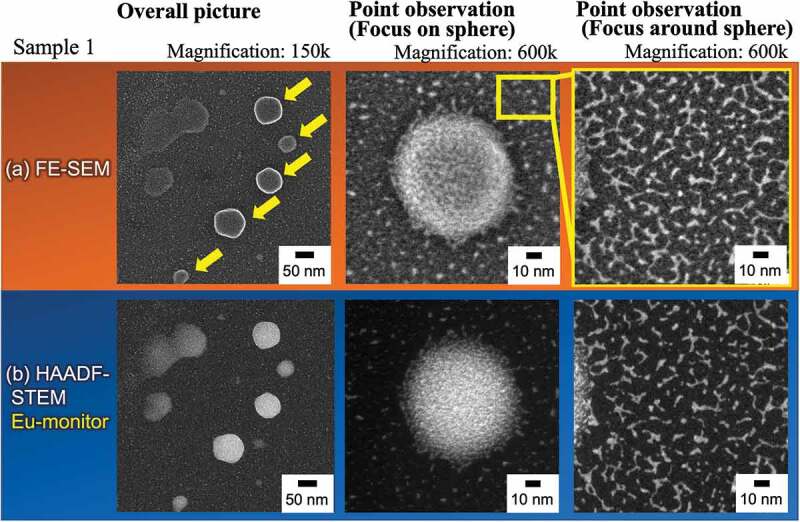
(a) FE-SEM images for Form 1 with various magnifications (×150 k and × 600 k). (b) High-angle annular dark field-scanning transmission electron microscopy (_HAADF-STEM_) images for Form 1. Right-hand-side images are enlarged views focusing on the yellow square in the top-center image.

Spherical high-brightness phosphors that can be fabricated at the nanoscale and are highly stable are particularly important candidates for practical use as nano phosphors for next-generation electronic devices. To elucidate the mechanism of the formation and spherical shape generation of Form 1, a detailed analysis was performed using SEM and STEM. Figures 7(a, b) show the FE-SEM images of Form 1 with magnifications of × 150 k and × 600 k, and the high-angle annular dark field-STEM (HAADF-STEM) images at the same position, respectively. As focus images, the right-hand-side images are enlarged views marked by the yellow square in the top-center image. For TEM sample preparation, after the reaction in the synthesis scheme of Form 1, the MeOH solution containing the LCPCs nano-seized spheres was dipped on a carbon grid, and the rapidly dried sample was observed. Spherical particles with an average diameter of approximately 100 nm and linear particles with an average diameter of approximately 15–25 nm were observed in the low-magnification image, and the HAADF analysis revealed that the two types of compounds included Eu. The EDX mapping for each main element was also roughly consistent, as shown in SI Figure S6. DLS measurements of the particle size distribution of Form 1 immediately after the synthesis reaction for 0–5 min (see SI, Figure S7) found that nanoparticles with the sizes of several tens of nanometers were dispersed in MeOH. These particle sizes are in rough agreement with the particle size observed in Figure 7, that is, the point observation around the sphere.

**Figure 8.**
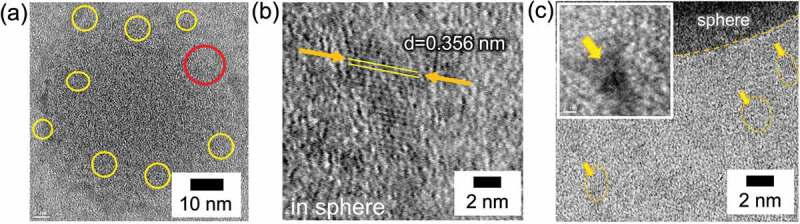
(a) Bright-field (BF)-STEM image of spherical particles for Form 1. (b) Enlarged view focused on the red circle in the left image. (c) BF-STEM image of the crystalline particles around the main sphere.

To explore the periodicity of the crystalline spherical particles, a bright-field (BF)-STEM image of spherical particles is shown in Figure 8(a), and a magnified image focused on the area marked by the red circle in Figure 8(a) is shown in Figure 8(b). The spherical particles with an average diameter of 80 nm were found to be aggregates of linear crystal particles with sizes of several tens of nanometers, and the particles were highly crystalline, allowing clear observation of the interplanar spacing of the lattice. In Figure 8(c), small linear particles around the large sphere can also be observed with clear interplanar spacings. We speculate that these particles are the smallest crystal units corresponding to the crystalline oligomers in macromolecules　(See in SI Figure S8). The coordination polymer formation of [Eu(hfa)_3_dpbp]_n_ is easily dominated by stacking reactions due to the strong intermolecular interactions between the planar conjugated organic ligands. Therefore, it is concluded that linear coordination oligomers were formed not in the extending direction of the rare earth complex, but rather in the intermolecular stacking direction.

**Figure 9.**
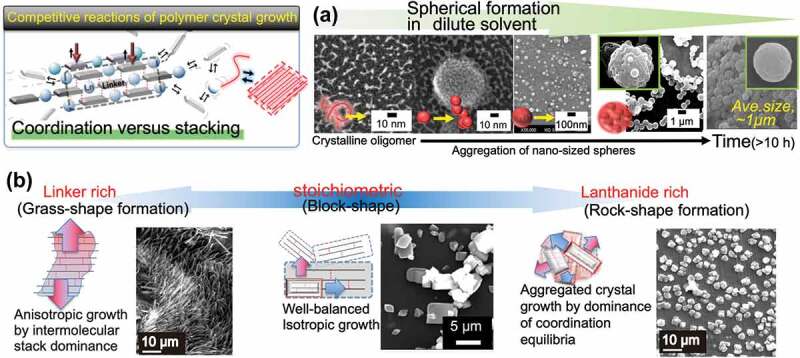
Representative two reaction schemes, coordination- and stacking-equilibrium for the formation of crystalline [Eu(hfa)_3_(dpbp)]_n_ coordination polymers. (a) Top-right images shows the proposed mechanism of particle formation. (b) Bottom images show morphological crystal changes under various synthesis conditions.

Figure 9 summarizes the morphological changes in the [Eu(hfa)_3_dpdp]_n_ LCPCs revealed in this study. The formation of crystalline [Eu(hfa)_3_(dpbp)]_n_ coordination polymers is controlled by two representative reaction schemes: coordination- and stacking-equilibrium. The proposed mechanism of spherical particle formation is shown on the top right in (A). In addition, the bottom three images in (B) summarize the morphological crystal changes under different stoichiometric conditions. As a first step, the reaction with the Eu precursor (*i.e*., mono nuclei [Eu(hfa)_3(_H_2_O)_2_]) and the coordinating linker ligand (*i.e*., dpbp) led to the formation of unstable polymer Eu-dpbp-Eu chains. Then, owing to the strong intermolecular forces, polymer chains form periodic structures by stacking and crystallization with adjacent molecules toward [11–1] direction. If the solute concentration in the reaction is low, such as for the formation of Form 1, because the surface energy of the polymeric crystals with a molecular size of several nanometers is high and the crystals are unstable, the crystals aggregate to reduce the surface energy as much as possible. Indeed, the 15–20 nm nanocrystals act as nuclei, and repeated aggregation of the particles gives rise to nano- to micro-sized spheres (*Ave*.~1.0 μm) with passing reaction time, as shown in (A)-process. In fact, the similar size of nanocrystals was observed in all of TEM images of the Form 1 series with different reaction times (~0, 1, 5, 6, and 10 h). Advantageously, the average size of the agglomerated spheres grows larger with reaction time in closed system while maintaining thermal stability, ultimately resulting in micro-sized bead spheres. In addition, the obtained spherical particles (*i.e*., Form 1 series) exhibited well dispersibility even in insoluble solvents, such as non-toxic alcohol. On the other hand, when the two equilibrium reactions were well-balanced, insoluble block-like homogeneous single crystal grains were obtained in the stoichiometric composition in (B)-process. When the amount of the linker was twice larger than the amount consistent with the crystal stoichiometry, a grass-like morphology oriented in the [11–1] direction was obtained due to anisotropic crystal growth. In contrast, when the amount of the Eu precursor was twice as large as the stoichiometric amount, a rock-like crystal morphology with high thermal stability was obtained.

### 3.4. Spherical crystalline [Eu(hfa)_3_dpdp]_n_ polymers in PMMA solid media for red-LEDs applications

**Figure 10.**
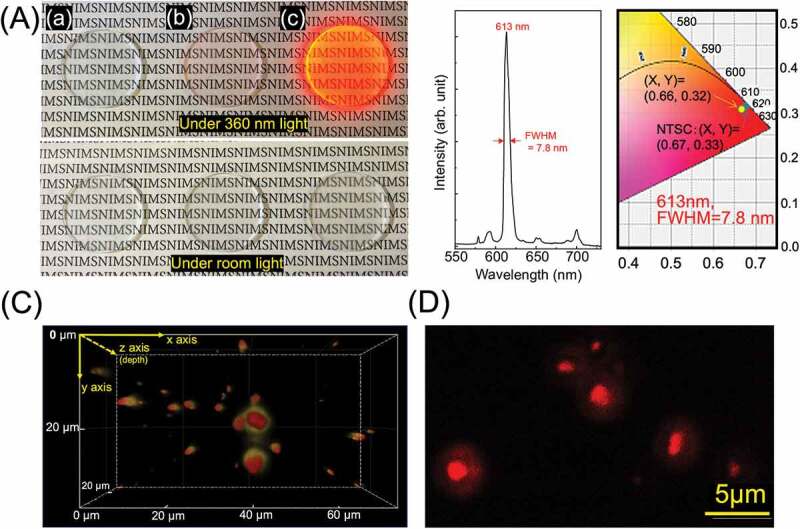
(A) Sample appearances, (a) no additive of LCPCs, (b) Standard and (c) from 1–2, under sunlight and 360 nm UV-light. (B) PL spectrum and CIE 1931 chromaticity coordinates of Form 1–2 in PMMA. (C) three-dimensional confocal fluorescent image in PMMA, and (D) is an enlarged fine two-dimensional fluorescent image of localized area.

Many of practical phosphor series, such as quantum dots and ceramic phosphors, are not used alone but are dispersed in a binder such as silicone, organic polymer or ink paint resins. Crystalline [Eu(hfa)_3_dpdp]_n_ phosphor is almost insoluble in ketonic or alcoholic solvents due to its high crystallinity and chemical stability, and even if dissolved, it would decompose. Therefore, its low dispersibility is a big issue when considering the phosphor production of transparent inks and sheets for next-generation μ-LED or OLED applications. Here, we investigated the medium dispersibility of morphology-controlled [Eu(hfa)_3_dpdp]_n_. The selected dispersion medium is a poly-methyl methacrylate (PMMA), which is actually used as the quantum dots or inorganic phosphor sheet coated on a polyethylene terephthalate (PET) film. A detailed fabrication scheme is shown in SI (description, Table S1, and Figure S9) The crystalline LCPCs [Eu(hfa)_3_dpbp]_n_, Standard and spherical Form 1–2 (average size at~1.0 μm, See in Figure S2), were each dispersed in MMA, and PMMA containing LCPCs was prepared by thermal polymerization. PMMA without LCPCs was also prepared as a reference. Figure 10(A) shows the appearance of the cut and polished samples, (a)no additive of LCPCs, (b) Standard and (c) From 1, under sunlight and UV light. All samples showed a transparent appearance similar to (a) no additive, but only (c) gave strong luminescence under 360 nm UV light. When polymer polymerization of MMA was carried out using an insoluble Standard LCPCs, it was found to be difficult to disperse due to sedimentation　in MMA (see in Figure S7). In contrast, with Form 1 LCPCs, the MMA solidified while maintaining high transparency. As a one of the photophysical properties, the PL spectrum and local CIE 1931 chromaticity coordinates is shown in Figure 10(B). Line emission peaked at 613 nm was observed, and the FWHM of the spectrum was 7.8 nm, which almost agreed with that of Form 1 powder. The decay lifetime (*τ*_obs_) changed slightly from 780 µs for Form 1 powder to 740 µs for in PMMA, and the internal quantum efficiency (*Φ*_Ln_) was estimated to 75%. The red coordinate point (x, y) calculated from the spectrum is (0.66, 0.32), which is comparable to the NTSC standard (0.67, 0.33)). To clarify the distribution state of spherical LCPCs (Form 1) in PMMA, three-dimensional distribution image measurement is performed on two different scales using a confocal fluorescence microscope. Figure 10(C) is the overall three-dimensional image, and (D) is an enlarged two-dimensional image of localized area. Fluorescent sphere particles with an average size diameter of 1 μm were partially aggregated in PMMA, however most of them were uniformly dispersed. In the next advanced stage of nano-phosphor materials, we expect that lanthanide coordinated phosphors with highly luminous and thermally stable, and easy morphology control, will be remarkable new phosphor materials. Therefore, this progress in the field of the design of new materials using crystalline coordination polymers that will play an important role in next-generation phosphor materials is expected to contribute to the improvement in structural selectivity and optical functionality.

## 4. Conclusion

We demonstrated the tailorability of the morphology of [Eu(hfa)_3_(dpbp)]_n_ LCPCs with high thermal stability obtaining nano- and micro-sized spheres, grass-shaped, block-type, and rock-shaped [Eu(hfa)_3_(dpbp)]_n_ LCPCs. In particular, the formation of spherical particles composed of nanosized crystalline polymers shows size-tunability through simple reaction time dependence. Additionally, excellent photophysical properties were achieved. Morphology-tailorable LCPSs are promising materials for next-generation nanophosphors such as micro-LEDs or OLEDs that require thermal durability.

## Supplementary Material

Supplemental Material
