# Supplementary material for: Structural metamorphosis and photophysical properties of thermostable nano- and microcrystalline lanthanide polymer with flexible coordination chains
Source: Sci Technol Adv Mater. 2023 Mar 3;24(1):2183711. doi: 10.1080/14686996.2023.2183711 (PMC9987761; doi:10.1080/14686996.2023.2183711)
Supplement: Supplemental Material [file TSTA_A_2183711_SM1724.docx]

Supporting Information

**Structural metamorphosis and photophysical properties of thermostable nano- and microcrystalline lanthanide polymer with flexible coordination chains**

Takayuki Nakanishi,^1^* Yuichi Hirai,^2^ Jian Xu,^2^ Takashi Takeda,^1^ Shunsuke Watanabe,^3^

Atsuo Yasumori,^3^ Shou Hakamada,^4^ Yuichi Kitagawa,^4, 5^ Yasuchika Hasegawa,^4, 5^

^1^ Research Center for Functional Materials, National Institute for Materials Science (NIMS),

- 1. Namiki, Tsukuba, Ibaraki, 305-0044, Japan.

^2^ International Center for Young Scientists (ICYS), National Institute for Materials Science (NIMS),

- 1. Namiki Tsukuba Ibaraki, 305-0044, Japan.

1. Department of Materials Science and Technology, Tokyo University of Science,

6-3-1 Niijuku, Katsushika-ku, Tokyo, 125-8585, Japan.

^4^ Faculty of Engineering, Hokkaido University,

Kita-13 Nishi-8 Kita-Ku Sapporo Hokkaido, 060-8628, Japan

^5^ Institute for Chemical Reaction Design and Discovery (WPI-ICReDD), Hokkaido University,

Kita-21 Nishi-10 Kita-Ku Sapporo Hokkaido, 001-0021, Japan

**Synthesis**

**Materials**

Europium acetate monohydrate (99.9%), n-BuLi (in n-hexane, 1.6 M), and hydrogen peroxide were purchased from Kanto Chemical Co., Inc. 2,5-Dibromothiophene, 3,4-ethylenedioxythiophene, and chlorodiphenylphosphine were obtained from Tokyo Chemical Industry Co., Ltd. All other chemicals and solvents were reagent grade and were used without further purification.

**Preparation of** **tris(hexafluoroacetylacetonato)europium dihydrate [Eu(hfa)_3_(H_2_O) _2_]**

The preparation of [Eu(hfa)_3_(H_2_O)_2_] was based on our previous report.^[1]^ Europium acetate n-hydrate (2.0 g, 5.8 mmol, Weighing was calculated by molar mass without water ((CH_3_COO)_3_Eu, m.w. = 329.09 g/mol)) was dissolved in distilled water (30 mL). Hexafluoroacetylacetone (4.0 g, 19 mmol) was added dropwise to the solution and let stirred for 3 h at room temperature to form pale yellow precipitates. The reaction mixture was filtered and washed with distilled water and chloroform. The resulting powder was used without further purification for the next step.

[Eu(hfa)_3_(H_2_O)_2_]: Yield 4.1 g (89%). IR (KBr): 1650 (st, C=O), 1258-1145 (st, C-F) cm^-1^. Anal. Calcd for C_15_H_7_EuF_18_O_8_: C, 22.27; H, 0.87%. Found: C, 22.12; H, 1.01%

**Preparation of 4,4’-bis(diphenylphosphoryl)biphenyl [dpbp]**

4,4’-Bis(diphenylphosphoryl)biphenyl was synthesized according to the published our procedure [Main txt Ref in [11]]. A solution of n-BuLi (9.3 mL, 1.6 M hexane, 15 mmol), was added dropwise to a solution of 4,4’-dibromobiphenyl (1.9 g, 6.0 mmol) in dry THF (30 mL) at -80 ˚C. The addition was completed in ca. 15 min, during which time a yellow precipitate was formed. The mixture was allowed to stir for 3 h at -10 ˚C, after which PPh_2_Cl (2.7 mL, 15 mmol) was added dropwise at -80 ˚C. The mixture was gradually brought to room temperature and stirred for 14 h. The product was extracted with ethyl acetate, and the extracts were washed with brine three times and dried over anhydrous MgSO_4._ The solvent was evaporated, and the resulting residue was washed with acetone and ethanol several times. The obtained white solid and dichloromethane (ca. 40 mL) were placed in a flask. The solution was cooled to 0 ˚C and then 30% H_2_O_2_ aqueous solution (5 mL) was added to it. The reaction mixture was stirred for 2 h. The product was extracted with dichloromethane, and the extracts were washed with brine three times and dried over anhydrous MgSO_4_. The solvent was evaporated to afford a white powder. Recrystallization from dichloromethane gave white crystals of the titled compound.

[dpbp]: Yield: 1.1 g (33%). IR (KBr): 1120 (st, P=O) cm^-1^.^1^H NMR (400 MHz, CDCl_3_, 25 ^o^C) d 7.67–7.80 (m, 16H; P-C_6_H_5_, C_6_H_4_), 7.45–7.60 (m, 12H; P-C_6_H_5_, C_6_H4) ppm. MS (ESI) found: m/z = 555.2, calcd for C_36_H_29_O_2_P_2_: [M + H]^+^, 555.2. Anal. calcd for C_36_H_28_O_2_P_2_: C, 77.97%; H, 5.09%. Found: C, 77.49%; H, 5.20%.

**Preparation of [Eu(hfa)_3_(dpbp)]_n_**

The dpbp (0.40 g, 0.72 mmol) in 15 mL MeOH was added into the solution of Eu(hfa)_3_(H_2_O)_2_ (0.58 g, 0.72 mmol) in 10 mL MeOH. The solution was stirred in a closed system at 60 °C for 5 h, and then the obtained white precipitate was filtered and washed with cooled MeOH (~0 °C). The obtained white powder sample is named as “Standard.” ed MeOH (~0 °C). The obtained white powder sample is named as “Standard.” The other samples, named “Form 1, 2, and 3” were obtained by the same method, but using the solvent MeOH amounts, and the mixing ratios of the chemicals presented in Table 1. Form 1 was obtained as a transparent liquid; therefore, the sample was collected by centrifugation and by a rapid filtration technique using a nano-membrane filter.

**[Standard: [Eu(hfa)_3_(dpbp)]_n_]**: Yield 0.63 g (66%). IR: 1653 (st, C=O), 1255–1145 (st, C–F), 1140 (st, P=O) cm^–1^. ESI–Mass (m/z) = 1121. [Eu(hfa)_2_(dpbp)]^+^, 2447.15 [Eu_2_(hfa)_5_(dpbp)_2_]^+^. Anal. Calcd for [C_51_H_31_EuF_18_O_8_P_2_]_n_: C, 46.14; H, 2.35%. Found: C, 45.59; H 2.49%.

**[Form 1]**: Yield N/A g (N/A%). IR:1653 (st,C=O), 1255–1145 (st,C–F), 1140 (st, P=O) cm^–1^.

**[Form 2]**: Yield 0.54g (56%). IR: 1653 (st, C=O), 1255–1145 (st, C–F), 1140 (st, P=O) cm^–1^.

**[Form 3]**: Yield 0.72g (75%). IR: 1653 (st, C=O), 1255–1145 (st, C–F), 1140 (st, P=O) cm^–1^.

**Figure S1.** Relative relationship between elongation direction [101] and crystal orientation direction [11-1] of [Eu(hfa)_3_dpbp]_n_ crystal, which illustrated using crystal information files.

1.
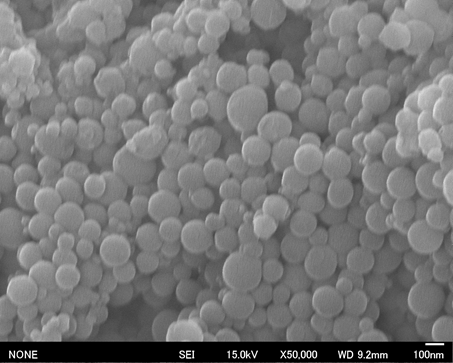

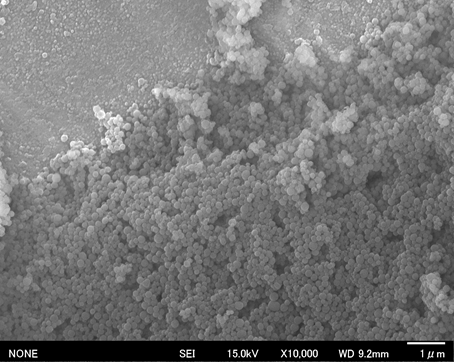
Reaction time 6 h (Form 1-1 sample)


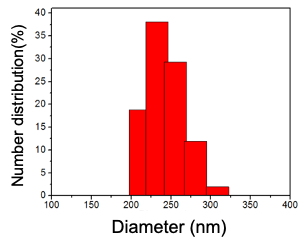


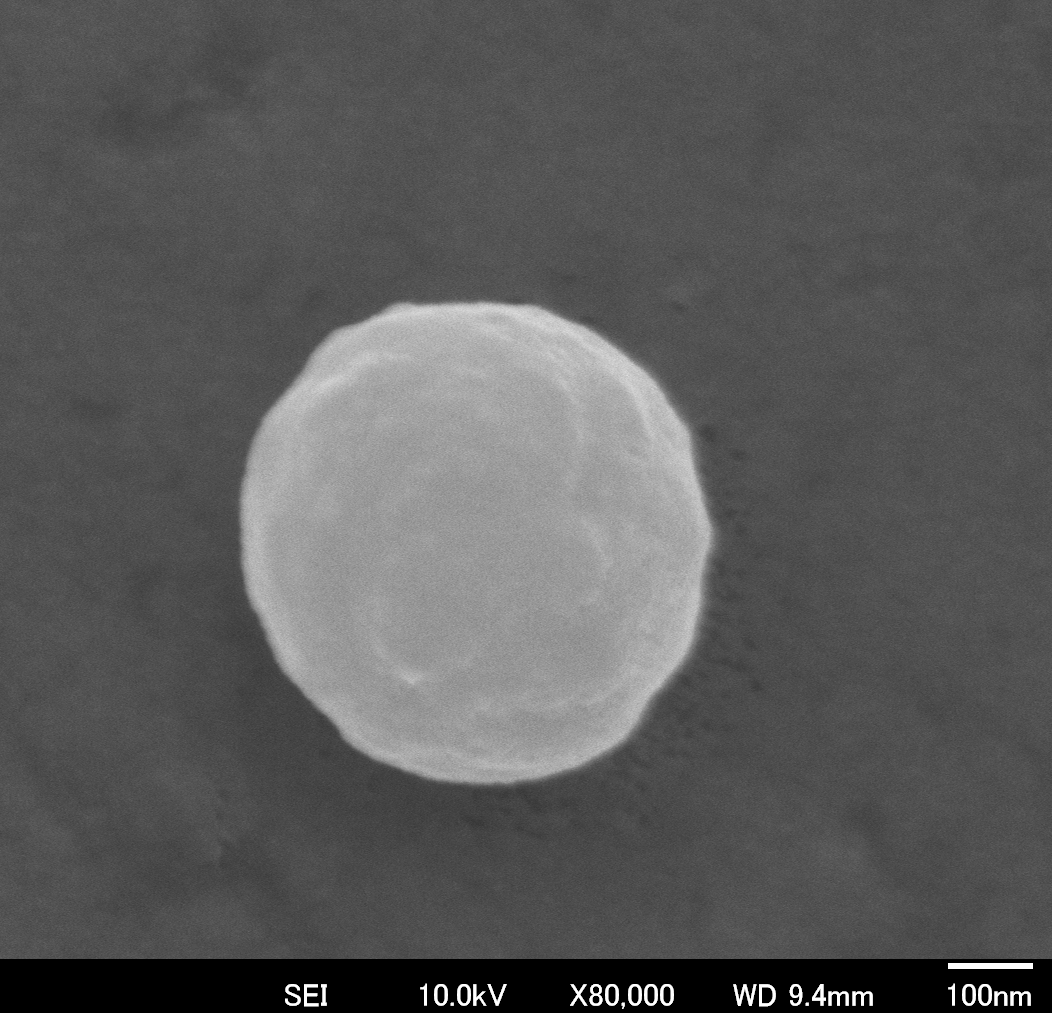

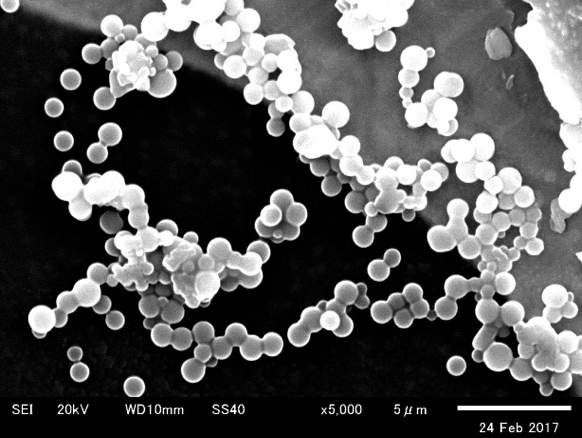
(b)Reaction time 10 h (Form 1-2 sample)


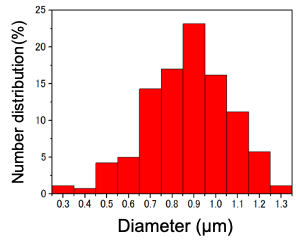


Figure S2

**Figure S2**. FE-SEM images and DLS size distributions of Form 1 series samples, which prepared under dilute solvent, 250 mL MeOH, reaction time **6 h (Form 1-1)**, and **10 h (Form 1-2))**.


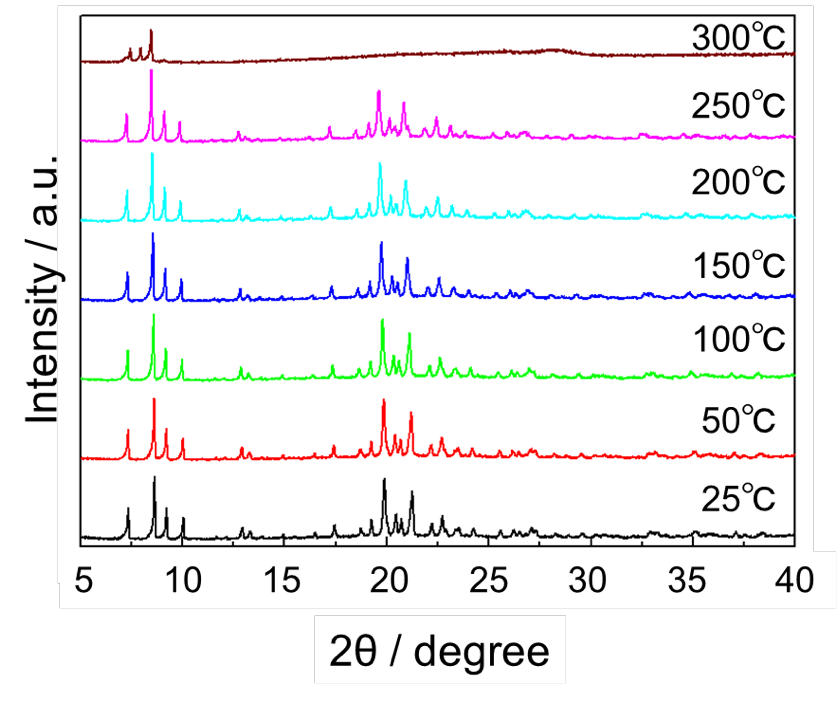


**Figure S3.** XRD patterns under various temperature (25-300°C, in-site measurement) of [Eu(hfa)_3_(dpbp)]_n_ (Standard).


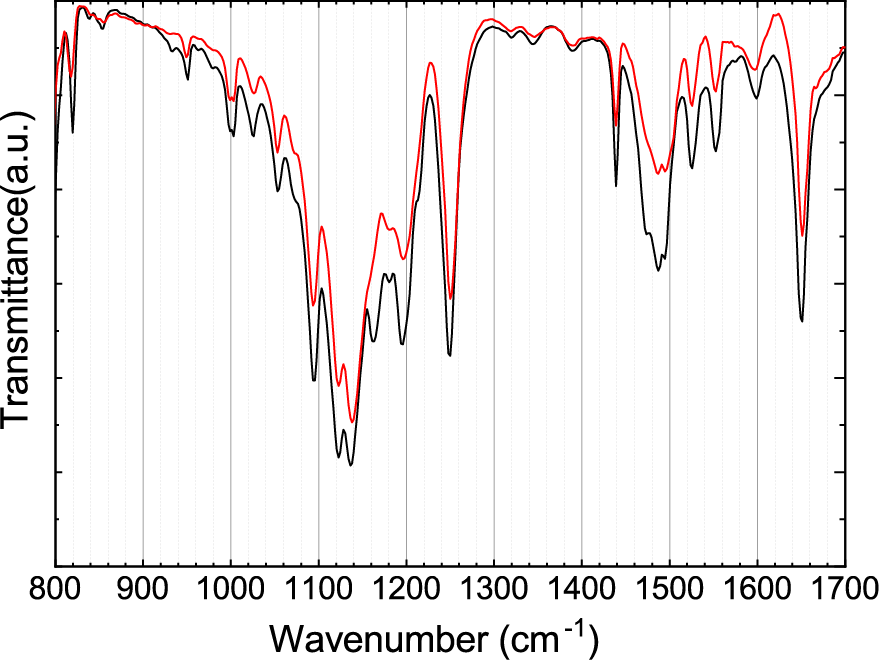


**Figure S4.** FT-IR spectra of [Eu(hfa)_3_(dpbp)]_n_ (standard) at room temperature, before and after heating at 300°C for 30 min.

**Figure S5.** PL spectra of [Eu(hfa)_3_(dpbp)]_n_: (a) Standard, (b) Form 2, and (c) Form 3 as solid-state. Excitation wavelength is at 365nm.


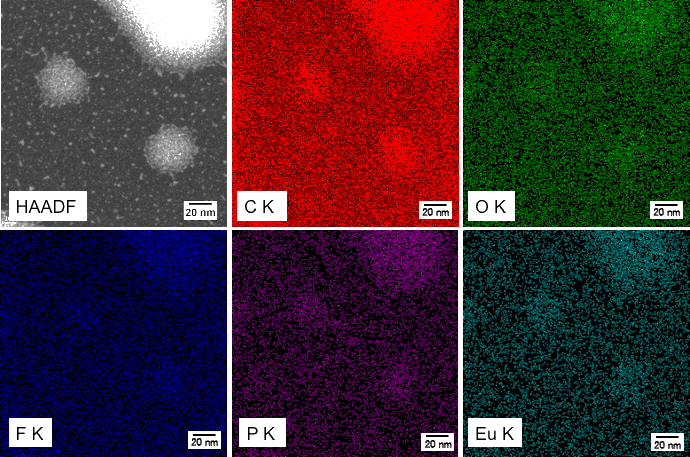


**Figure S6.** TEM-EDX mapping for Eu, F, P, O and C. HAADF image also shown in Figure.

**Figure S7.** DLS measurement for (b) From 1’ (as-prepared liquid, reaction time >0h). LB-500 Dynamic Light-Scattering Particle Size Analyzer, Horiba. co, Japan


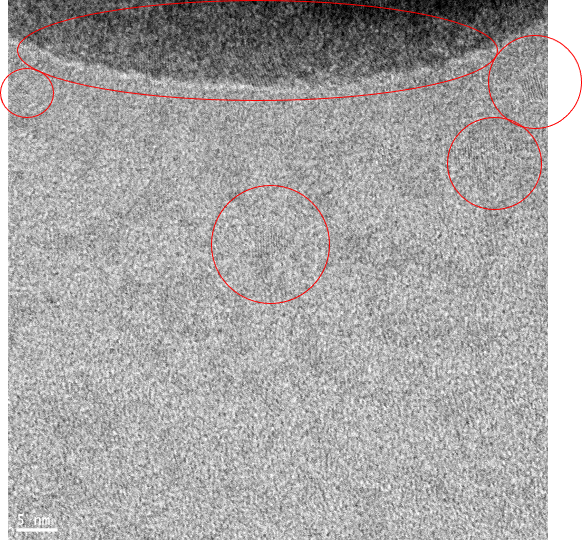


**Figure S8.** STM-image focused on around sphere. This is an original data for Figure 8(c).

**Preparation of [Eu(hfa)_3_(dpbp)]_n_ in poly methyl methacrylate[PMMA]**

The obtained crystalline lanthanide coordination compounds, [Eu(hfa)_3_dpbp]_n_, Standard and Form 1, were each dispersed in methyl methacrylate (MMA), and PMMA containing LCPCs was prepared by thermal polymerization. Eu-LCPCs (22 mg, 0.33wt%) were dispersed in MMA (7 mL, 6.6 g) and placed in a test tube. At -40°C, 2, 2'-azobisisobutyronitrile (AIBN) was added to be 0.125wt% relative to the amount of MMA. Thermal polymerization was carried out by performing nitrogen substitution and reacting at 50°C for 2 hours, followed by reacting at 60°C for 78 hours. Table 1S shows the amounts of Eu, MMA, and AIBN for PMMA preparation. A reaction scheme is also shown below.

Table S1. Synthesis conditions of the LCPCs-PMMA composite.

|  | Concentration of LCPCs g(wt%) | MMA  mL(g) | Addition amount of AIBN g(wt%) |
| --- | --- | --- | --- |
| (a)No additives | 0.00(0.00) | 5(4.4) | 0.012(0.125) |
| (b)Standard | 0.22(0.33) | 7(6.6) | 0.018(0.125) |
| (c)Form 1 | 0.22(0.33) | 7(6.6) | 0.018(0.125) |


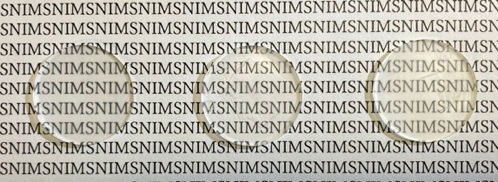


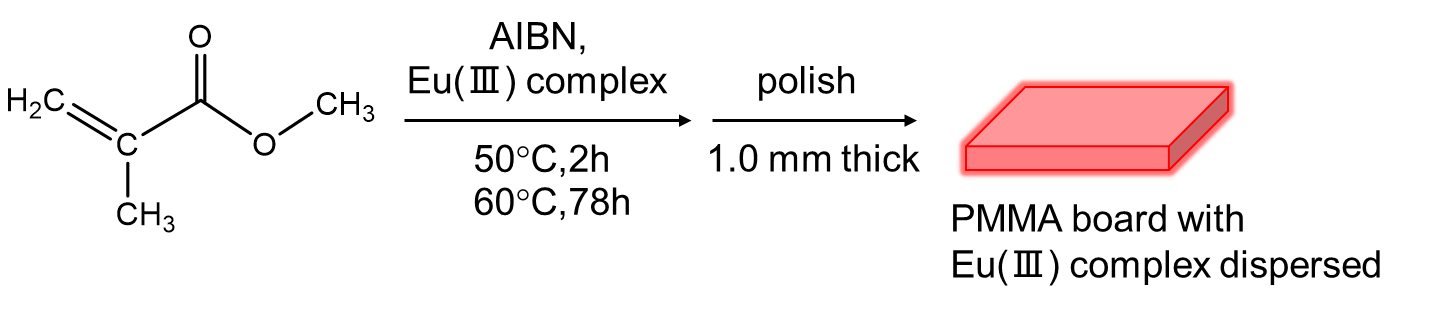


(c)

(b)

(a)

**Figure S9.** [Eu(hfa)_3_dpdp]_n_ dispersed in MMA before thermal polymerization. Images (a) and (c) shows the MMA liquid using Standard and Form 1 under sun light. Images (b) and (c) is the MMA liquid under UV-light (λ_ex_=360 nm).

**References**

[1] Y. Hasegawa, M. Yamamuro, Y. Wada, N. Kanehisa, Y. Kai, S. Yanagida, J. Phys. Chem. A 2003, 107, 1697–1702.
